# Three years of COVID-19 in children that attend the Mexican Social Security Institute's 1,350 child day-care centers, 2020–2023

**DOI:** 10.3389/fped.2023.1292629

**Published:** 2024-01-04

**Authors:** Libny Martínez-Valdez, Vesta L. Richardson, Aurora Bautista-Márquez, Martín Alejandro Camacho Franco, Vicente Cruz Cruz, Mauricio Hernández Ávila

**Affiliations:** Dirección de Prestaciones Económicas y Sociales del Instituto Mexicano del Seguro Social, Mexico City, Mexico

**Keywords:** COVID-19, child day-care centers, hospitalizations, deaths, Mexico, children

## Abstract

**Background:**

Studies have suggested that children are less likely than adults to develop COVID-19; however, with the emergence of SARS-CoV-2 variants, hospitalization and death due to this cause have increased among the youngest ones.

**Methods:**

Retrospective, descriptive analytical study of the COVID-19 cases, hospitalizations and deaths occurred in children under five years who attended in Child Day-Care Centers (*Centros de Atención Infantil-*CAIs*)* of the Mexican Social Security Institute (IMSS) from 20th July 2020 to 31st March 2023. Results were compared with Mexico's and the US's national-level data. Incidence, attack (children and workers) and mortality rates were estimated. The risks of getting sick, being hospitalized and dying due to COVID-19 were calculated by year.

**Results:**

There were 4,369 COVID-19 cases among children from IMSS CAIs; 67 (1.5%) required hospitalization and only two deaths were reported (0.04%). Both at IMSS CAIs and at a national level in Mexico and the US, the highest incidences of COVID-19 among children under five years occurred during Omicron prevalence. The attack rate among workers (32.93%) was higher than children (4.99%). Hospitalization and mortality rates in the US decreased since the anti-COVID 19 vaccine was introduced in children older than six months, unlike the rates in Mexico, where the vaccine for this age group was not available. By the year 2020, the children that attended the IMSS CAIs were 77.3% less likely to be hospitalized; 80.9% in 2021, 93.2% in 2022, and 77.7% by March 2023, compared to same age children in Mexico. In 2021, the children that attended IMSS CAIs were 90.6% less likely to die due to COVID-19, and by March 2023, this likelihood was 34.3% lower than the rest of children in this age group in Mexico.

**Conclusions:**

Children that attended IMSS CAIs had a smaller risk of hospitalization and death due to COVID-19. However, the high rates of hospitalization and death due to SARS-CoV-2 in children under five years in our country point to the need and urgency of vaccination against this virus in this age group, as well as of the adherence to strict detection and medical referral protocols.

## Introduction

The Mexican Social Security Institute (*Instituto Mexicano del Seguro Social* – IMSS) is the biggest social security institution in the country. It provides care to close to 200 thousand children between 43 days and four years of age in a little more than 1,300 Child Day-Care Centers (*Centros de Atención Infantil* – CAIs) with about 55,000 workers. The centers are distributed in the 32 federal entities.

On 11 March 2020, the World Health Organization (WHO) declared COVID-19 as a pandemic. In Mexico, as in other countries, initially the shutting down of Child Day-Care Centers and schools was mandated in March 2020 as a prevention measure in the face of the sanitary emergency (1). However, as of July 2020, the IMSS CAIs gradually resumed their activities, and they implemented the “Guidelines and Action Plan of IMSS Childcare Facilities in the Face of the New Coronavirus” (2). These established the prevention and control measures of SARS-CoV-2 transmission, such as the use of face masks and protective masks, frequent handwashing, use of 70% alcohol hand sanitizer gel, reduced groups of children, three daily rounds of intentional search of cases, mandatory measurement of body temperature, home confinement of children and workers with infectious symptomatology and social distancing measures, shutting down of care rooms and immediate medical referrals of children and workers with respiratory symptoms for evaluation.

Several studies have suggested that children are less likely than adults to develop an infection due to SARS-CoV-2, they are less infectious, most display mild symptoms or they are even asymptomatic (3–6). Moreover, susceptibility to the infection seems to increase with age (6–8), and transmission rates of SARS-CoV-2 are low in schools and in child-care spheres (7, 9). There are published studies that suggest that educators play an important role in the transmission mechanism in schools (10). It is also known that serious disease and hospitalization due to COVID-19 are more common among older adults, but children can also be affected (11–13).

It has been estimated that between 1% and 20% of pediatric infections due to SARS-CoV-2 require hospitalization, although this can be an overestimation, since many asymptomatic or minimally symptomatic pediatric infections can go undiagnosed (6). Regarding the pediatric age group that more frequently requires hospitalization due to COVID-19, the Centers for Disease Control and Prevention (CDC) analyzed the information from six hospitals in the US and reported that, during the period from July to August 2021, 713 children under 17 years of age had been hospitalized with COVID-19, out of which 24.7% were under one year of age, 17.1% were between 1 and 4 years old, 20.1% were between 5 and 11 years old, and 38.1% were between 12 and 17 years old (14).

A study in Mexico among children under 18 years of age with COVID-19, from March 2020 to September 2021, reported that children under one year with COVID-19 in our country had a higher risk of hospitalization and death than older children. The lethality rates were 0.2% for children between 10 and 17 and between 6 and 9 years of age, 0.8% for children between 1 and 5 years of age, and 4.2% for children under one year of age (15). On the other hand, a Brazilian study reported that in 2021, that country had a lethality rate due to COVID-19 of 3.7% for children under one year of age, 2.1% for children between 1 and 4 years of age, 4.2% for the group between 5 and 11 years of age, 9% for teen-agers between 12 and 14 years of age, and 9.8% in those between 15 and 18 years of age (16).

It was during the emergence of Omicron when the increase of seroprevalence and hospitalization burden in children under five years of age was observed in the US; the data were similar or higher than those of other pediatric diseases preventable through vaccination. Therefore, as of 18 June 2022, the CDC recommended anti-COVID-19 vaccination for children between 6 months and 4 years of age (17, 18). In contrast, up to the date when this study was conducted, Mexico had not implemented vaccination in children under five years of age.

The objective of this study is to describe the incidence, characterization and risk of getting sick, being hospitalized and/or dying due to COVID-19 among the children cared for at the IMSS CAIs during the sanitary emergency and compare them with the data at a national level in Mexico and in the US.

## Methods

It is a retrospective, descriptive analytical study of the COVID-19 cases, hospitalizations and deaths occurred in children between 43 days and four years and 10 months of age who attended IMSS CAIs from 20th July 2020 to 31st March 2023, and in children under five years of age at a national level in Mexico and in the US.

In Mexico, since the beginning of the pandemic, the “Standard guidelines for the epidemiological surveillance and viral respiratory disease laboratory” was used to diagnose COVID-19 cases (19). It establishes that epidemiological surveillance has to be carried out through 473 medical units called “Viral Respiratory Disease Health Monitoring Unit” (*Unidad de Salud Monitora de Enfermedad Respiratoria Viral* – USMER), taking samples from 100% of the suspected cases with mild symptoms and from 100% of the cases with severe symptomatology. In the remaining units (not USMERs), a sample is taken from 100% of the cases that meet the operational definition of severe acute respiratory infection and from 10% of the cases without severity. This information is recorded in an official open data system of the Ministry of Health. For the purpose of this analysis, the confirmed cases (by RT-PCR- or quick antigen test, by epidemiological clinical association and by expert ruling) of children under five years of age contained in the above-mentioned open data system, for the study period, were included (20).

The information on the confirmed COVID-19 cases in children and workers of IMSS CAIs from July 2020 to June 2021, was obtained from an Excel database built from the information on the cases and received via email. As of July 2021 and up to 31st March 2023, the information on the confirmed cases in children and workers of IMSS CAIs was obtained from the information system called “Epidemiological surveillance system in childcare facilities” (*Sistema de vigilancia epidemiológica de guarderías* – SVEG). This platform contains variables related to the child's identification data [name, age, sex, federal entity of residence and Unique Population Registry Key (*Clave Única de Registro de Población* – CURP) that the Mexican government grants its inhabitants], CAI identification data, date of symptom onset, type of care received (ambulatory or hospitalization), type of diagnostic test performed [real-time polymerase chain reaction (RT-PCR) or quick antigen test, both through nasopharyngeal exudate], date of hospitalization and, when applicable, date of death. In the case of the workers, the following variables were used: CURP, age, CAI identification data, date of symptom onset, type of diagnostic test performed (RT-PCR or quick antigen test, both through nasopharyngeal exudate). For the purpose of this study, the information of those cases that were positive by RT-PCR or quick antigen test, both for children and for workers at IMSS CAIs, was used. The information recorded in the above-mentioned system was extracted on an Excel 2010 (v14.0) database to be analyzed.

The enrollment, dropouts and attendance registers of children and workers at the IMSS CAIs were extracted from the Childcare Facilities Information and Administration System (*Sistema de Información y Administración de Guarderías* – SIAG). Mid-year population estimations (2020–2023) were obtained from the dynamic cubes of the Health Information General Direction at the Ministry of Health of the Mexican Government (21).

The information related to the cases, hospitalizations and deaths due to COVID-19 occurred in children under five years of age in the US for the study period was obtained from the CDC's COVID-19 data tracker (22).

Simple frequencies by sex, age, month and year of occurrence were estimated to characterize cases, hospitalizations and deaths by COVID-19 in children that attended IMSS CAIs and in children under five years of age at a national level.

The incidence of COVID-19 in children was estimated, both in the CAIs and at a national level, using as numerator the total of confirmed cases, and as denominators the average of children cared for at the CAIs by month during the study period and the mid-year (2020–2023) population estimation in children under five years of age in Mexico, respectively. Both results were multiplied by 100,000 children. The COVID-19 incidence rates by epidemiological week halfway through each month in children under five years of age in the US, published by the CDC, were used as reference.

The monthly COVID-19 attack rate was also estimated for children and workers that attended the CAIs during the study period. The number of confirmed cases (children or workers) was used as numerator, and the daily average attendance by children or workers to the CAIs for each month during the study period was used as denominator. The accumulated attack rate for the whole period study was also estimated, using the total accumulated cases as numerator, and the average monthly attendance to the CAIs during the study period as denominator. Likewise, the annual COVID-19 attack rate in children from the CAIs by federal entity was estimated, using the total cases by year by federal entity as numerator, and the average monthly attendance to the IMSS CAIs by year and federal entity as denominator. All the results were multiplied by 100.

The hospitalization and mortality rates due to COVID-19 were estimated in children under five years of age at a national level using the cases of hospitalization or death as numerator, and the mid-year (2020–2023) population estimation of children under five years of age in Mexico as denominator. Both results were multiplied by 100,000 children. The hospitalization rate due to COVID-19 was estimated in the children from the CAIs, using the COVID-19 cases in children that required hospitalization as numerator, and the average of children cared for by month during the same period as denominator; the result was multiplied by 100,000. The hospitalization and mortality rates by epidemiological week halfway through each month in children under five years of age in the US, published by the CDC, were also used as reference (22).

The ratio of children with COVID-19 that attend a CAI and were hospitalized or died was estimated by month for the study period. This was compared with the data at a national level.

From the confirmed COVID-19 cases at a national level obtained from the Mexican Government's Ministry of Health's official platform (20), the relative risk for the children cared of at the IMSS CAIs to get sick, be hospitalized or die due to COVID-19 was estimated using a two-by-two table. Moreover, using a *χ*^2^ test with a significance level of 5%, it was estimated whether the ratios of cases, hospitalizations and deaths by sex and age group displayed statistically significant differences during the study period.

## Results

In Mexico, during the study period, the official platform of the Ministry of Health reported 74,352 confirmed cases of COVID-19 in children under five years of age (20). During the same period, 4,369 confirmed cases of COVID-19 in children that attended the IMSS CAIs were registered in the SVEG; the daily average of care included 97,469 children between July and December 2020, 158,956 children in 2021, 186,789 children in 2022, and 189,918 children cared for daily during the first three months of 2023.

Out of the 74,352 confirmed cases of COVID-19 in children under five years of age reported in the official platform of the Ministry of Health, 2,929 (3.93%) had some comorbidity (asthma 0.9%, cardiovascular disease 0.7%, immunosuppression 0.6%, obesity 0.6%, high blood pressure 0.5%, diabetes 0.4%, chronic obstructive pulmonary disease 0.1% and chronic kidney disease 0.1%), 39,917 (54%) were male, and the age group with more cases corresponded to children between 13 and 24 months of age (26%). A total of 9 978 (13.4%) children under five years of age with COVID-19 required hospitalization, out of which 5,767 (58%) were male, and 3,300 (33%) of the hospitalized ones belonged to the age group between 43 days and 12 months old. There were 634 (0.85%) deaths; 339 (53%) were male and most of the 276 (44%) deceased corresponded to the group between 43 days and 12 months of age (Table 1).

**Table 1:** Characterization of the cases of COVID-19 in children under 5 years of age in Mexico, from July 2020 to March 2023.

National level Mexico	2020			2021			2022			2023^a^			Totals		
	Cases	Hospitalized	Deceased	Cases	Hospitalized	Deceased	Cases	Hospitalized	Deceased	Cases	Hospitalized	Deceased	Cases	Hospitalized	Deceased
	*N* = 5792	*N* = 1,212	*N* = 173	*N* = 23,847	*N* = 2,890	*N* = 223	*N* = 40,807	*N* = 4,915	*N* = 203	*N* = 3,906	*N* = 961	*N* = 25	*N* = 74,352	*N* = 9,978	*N* = 634
	100%	20.92%	2.98%	100%	12.10%	0.93%	100%	12%	0.49%	100%	24.60%	0.64%	100%	13.40%	0.85%
Sex															
Male	3,068	707	104	12,626	1,676	121	22,104	2,841	100	2,119	543	14	39,917	5,767	339
	53%^NS^	58%	60%	53%	58%	52%	54%	58%^NS^	49%	54%^NS^	57%^NS^	56%	54%	58%^NS^	53%
Female	2,724	505	69	11,221	1,214	112	18,703	2,074	103	1,787	418	11	34,435	4,211	295
	47%	42%	40%	47%^b^	42%^b^	48%	46%^b^	42%	51%	46%	43%	44%	46%^b^	42%	47%
Age															
43 days to 12 months	2,067	762	113	4,348	1,182	114	4,992	1,246	49	197	110	–	11,604	3,300	276
	36%^NS^	63%	65%	18%^NS^	41%^NS^	49%	12%	25%	24%	5%	11%		16%	33%	44%
13–24 months	1,036	165	29	5,232	745	66	11,313	1,737	91	1,937	586	20	19,518	3,233	206
	18%	14%	17%	22%	26%	28%	28%^NS^	35%	45%	50%^b^	61%^b^	80%	26%	32%^NS^	32%
25–36 months	880	109	13	4,640	406	21	8,469	887	34	713	130	3	14,702	1,532	71
	15%	9%	8%	19%	14%	9%	21%	18%	17%	18%	14%	12%	20%	15%	11%
37–48 months	857	96	10	4,609	312	18	7,938	577	16	538	65	1	13,942	1,050	45
	15%	8%	6%	19%	11%	8%	19%	12%	8%	14%	7%	4%	19%	11%	7%
49–58 months	952	80	8	5,018	245	14	8,095	468	13	521	70	1	14,586	863	36
	16%	7%	5%	21%^b^	8%	6%	20%^b^	10%	6%	13%^b^	7%	4%	20%^b^	9%	6%

NS, non-significant difference.

COVID-19 Open Data, General Direction of Epidemiology (Dirección General de Epidemiología – DGE), 17 April 2023 (20).

^a^ Confirmed cases are considered between 1st January and 31st March 2023.

^b^ *p* < 0.5, statistically significant difference at 5% between the ratio of cases, hospitalizations and deceased among the children at the CAIs and children under five years of age in Mexico.

The number of children attended in IMSS CAIs per year and age group is described in Table 2. Out of the 4,369 confirmed cases of COVID-19 in children from the IMSS CAIs, 1,307 (30%) were children between 25 and 36 months of age, 1,207 (28%) between 13 and 24 months of age, 941 (22%) between 43 days and 12 months of age, 871 (20%) between 37 and 48 months of age, and only 43 (1%) between 49 and 58 months of age. The highest ratio of cases in children under four years of age was significantly larger than among children in the same age groups at a national level. Confirmed cases were predominant among males (55%). Out of the total number of cases (4,369), 67 (1.53%) required hospitalization; out of them 36 (54%) were male and 47 (70%) were children under 24 months of age. Frequency of hospitalization tended to decrease as age increased. Two deaths, due to serious pneumonia by COVID-19, were reported in girls under one year of age with no comorbidity (Table 3).

**Table 2:** Children attended in the IMSS CAIs, from July 2020 to March 2023.

Age	2020	2021	2022	2023^a^	Totals
43 days to 12 months	89,986	317,659	401,269	101,145	910,059
13–24 months	160,918	518,891	604,020	150,839	1,434,668
25–36 months	191,847	615,092	704,192	178,736	1,689,867
37–48 months	141,748	453,173	528,298	138,113	1,261,332
49–58 months	172	1 110	1 610	464	3,356

Epidemiological surveillance platform in IMSS childcare facilities, 31st March 2023.

^a^ Between 1st January and 31st March 2023.

**Table 3:** Characterization of the cases of COVID-19 in children from the IMSS CAIs, from July 2020 to March 2023.

IMSS CAIs	2020			2021			2022			2023^a^			Totals		
	Cases	Hospitalized	Deceased	Cases	Hospitalized	Deceased	Cases	Hospitalized	Deceased	Cases	Hospitalized	Deceased	Cases	Hospitalized	Deceased
	*N* = 21	*N* = 1	–	*N* = 1,043	*N* = 25	*N* = 1	*N* = 3,072	*N* = 27	–	*N* = 233	*N* = 14	*N* = 1	*N* = 4,369	*N* = 67	*N* = 2
	100%	4.76%		100%	2.39%	0.09%	100%	0.87%		100%	6%	0.42%	100%	1.53%	0.04%
Sex															
Male	11	–	–	579	16	–	1,687	15	–	118	5	–	2,395	36	–
	52%			56%^b^	64%^b^		55%^b^	56%		51%	47%		55%^b^	54%	
Female	10	1	–	464	9	1	1,385	12	–	115	9	1	1,974	31	2
	48%^NS^	100^NS^		44%	36%	100%	45%	44%^NS^		49%^NS^	53%^NS^	100%	45%	46%^NS^	100%
Age															
43 days to 12 months	4	–	–	173	8	1	681	8	–	83	11	1	941	27	2
	19%			17%	32%	100%	22%^b^	30%^NS^		36%^b^	79%^b^	100%	22%^b^	40%^NS^	100%^NS^
13–24 months	4	–	–	314	9	–	836	10	–	53	1	–	1,207	20	–
	19^NS^			30%^b^	36%^NS^		27%	37%^NS^		23%	7%		28%^b^	30%	
25–36 months	8	–	–	314	5	–	924	5	–	61	2	–	1,307	12	–
	38%^b^			30%^b^	20%^NS^		30%^b^	19%^NS^		26%^b^	14%^NS^		30%^b^	18%^NS^	
37–48 months	5	1	–	229	3	–	603	4	–	34	–	–	871	8	–
	24%^NS^	100%^NS^		22%^b^	12%^NS^		20%^NS^	15%^NS^		15%^NS^			20%^NS^	12%^NS^	
49–58 months	–	–	–	13	–	–	28	–	–	2	–	–	43	–	–
				1%			1%			1%			1%		

NS, non-significant difference.

Epidemiological surveillance platform in IMSS childcare facilities, 31st March 2023.

^a^ Confirmed cases are considered between January 1st and March 31st 2023.

^b^ *p* < 0.5, statistically significant difference at 5% between the ratio of cases, hospitalizations and deceased among the children at the CAIs and children under five years of age in Mexico.

At the IMSS CAIs, the report of COVID-19 cases among the children, remained under 33 monthly cases during the first eleven months after the service was resumed, even when the service was yet to be reestablished in only three federal entities (108 accumulated cases from July 2020 to June 2021). However, the highest peaks of cases were observed during the period when the Omicron variant (BA.1 and BA.2/5) prevailed (915 cases in January 2022 and 783 cases in July 2022).

**Figure 1:**
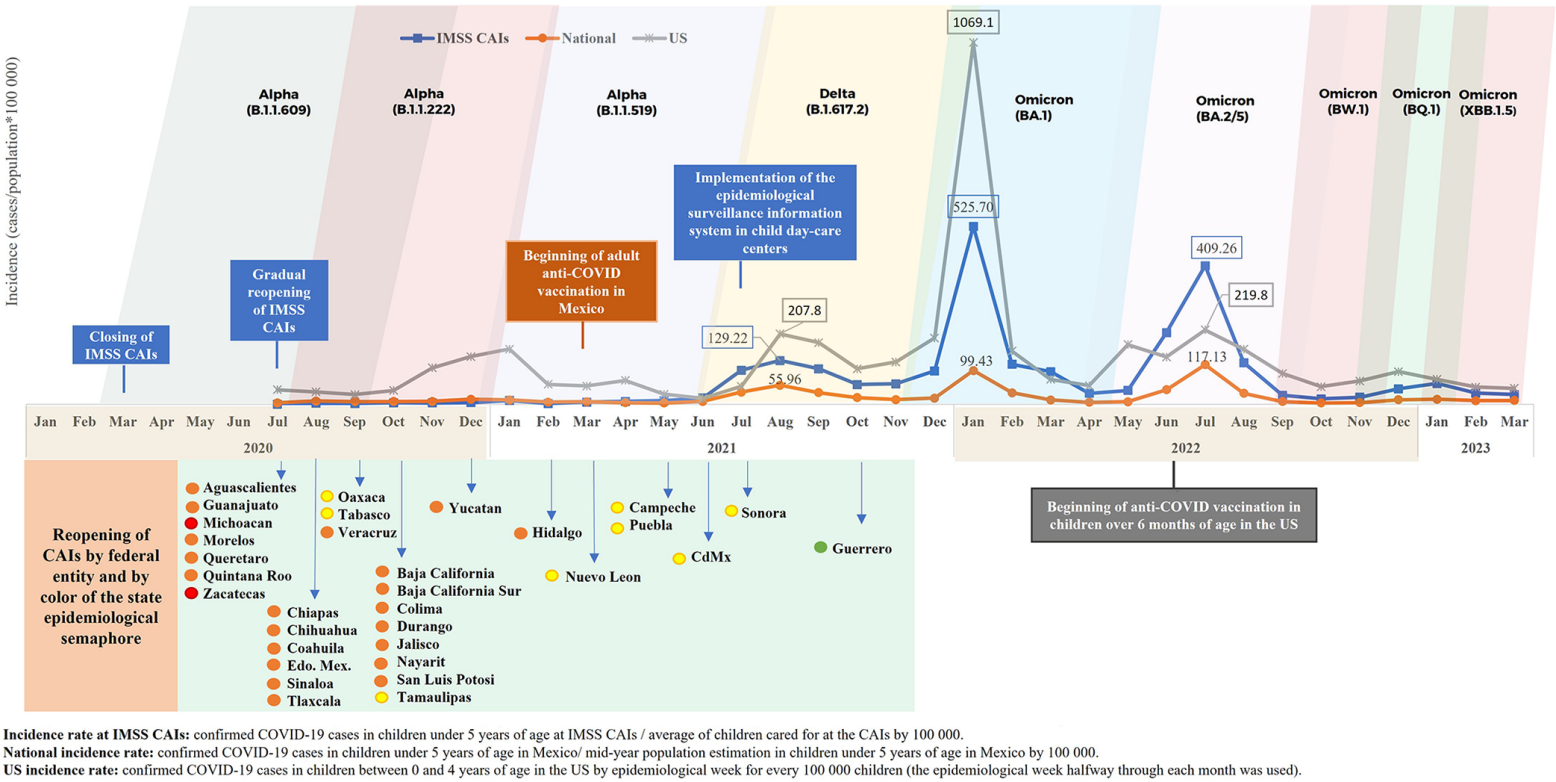
Incidence of COVID-19 in children from the IMSS CAIs, at a national level in Mexico and in the US, from July 2020 to March 2023. Sources: Epidemiological surveillance platform in IMSS childcare facilities, March 31st, 2023. Childcare Facilities Information and Administration System (*Sistema de Información y Administración de Guarderías* - SIAG). COVID-19 Open Data, General Direction of Epidemiology (*Dirección General de Epidemiología* - DGE), April 17th, 2023 (20). Mid-year (2020–2023) population estimation in children under five years of age in Mexico, General Direction of Health Information, Oct 2022 (21). COVID data tracker, US Centers for Disease Control and Prevention (CDC), June 10th, 2023 (22). COVID-19 variants in 2020–2023 (23, 24). //Edo. Mex: State of Mexico; CdMx: Mexico City.

Both at the IMSS CAIs and at a national level in Mexico and the US, the highest incidences occurred during the third quarter in 2021 (prevalence of Delta variant), in December 2021 and the first bimester in 2022 (prevalence of Omicron BA.1), between May and September 2022 (prevalence of Omicron BA.2/5) and at the end of 2022 and beginning of 2023. Between October 2020 and February 2021, an incidence peak, caused by variants Alpha B.1.1.222 and Alpha B.1.1.519, was reported in the US, but it was not observed in Mexico. The incidence rate was higher in the US during the whole study period, except from June 2022 onward (Figure 1).

**Figure 2:**
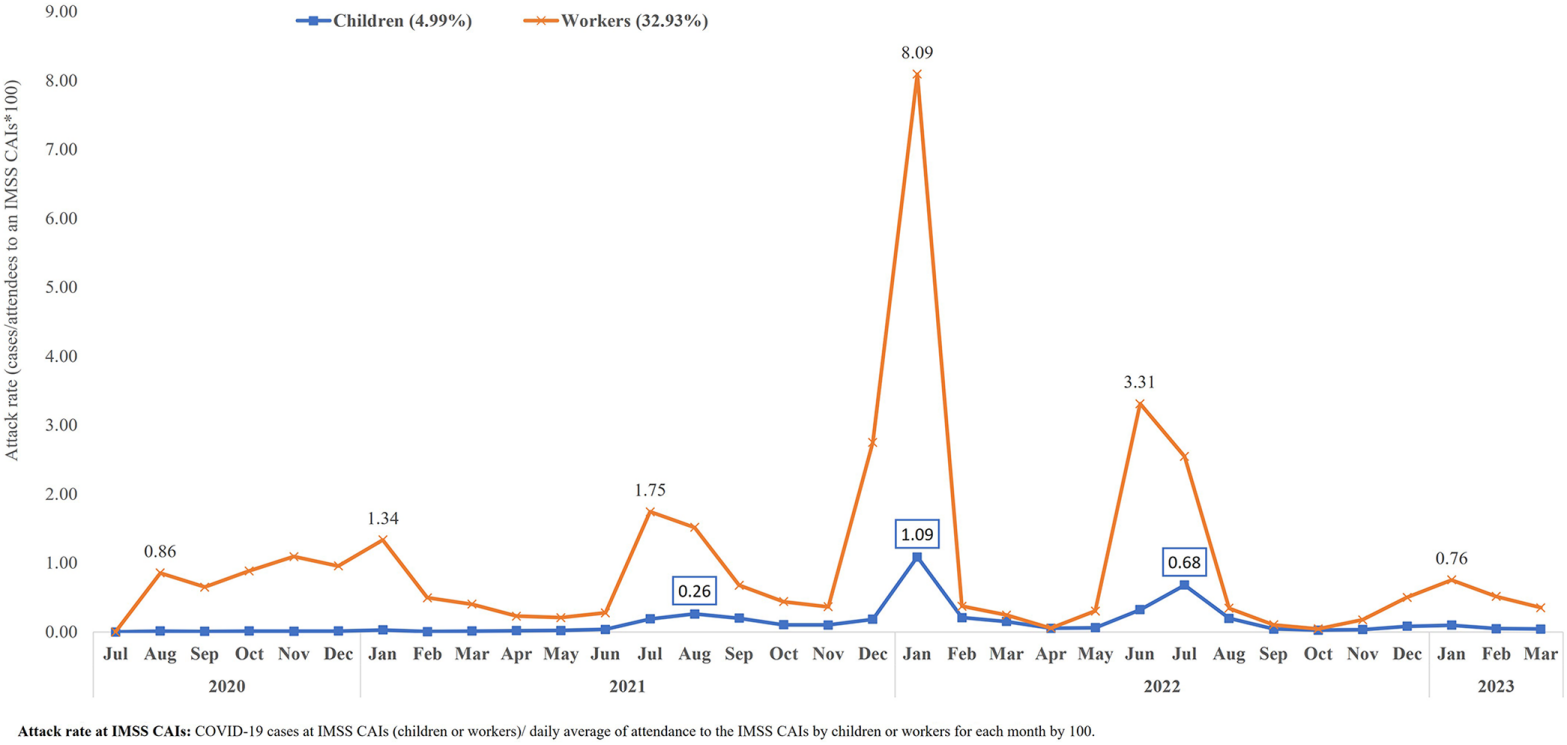
COVID-19 attack rate in children and workers at the IMSS CAIs, from July 2020 to March 2023. Source: Epidemiological Surveillance Platform in IMSS Childcare Facilities. March 31st, 2023. Childcare Facilities Information and Administration System (SIAG).

The accumulated attack rate in children that attended the CAIs during the study period was substantially lower (4.99%) compared to the attack rate among the workers (32.93%) in these facilities (Figure 2).

**Figure 3:**
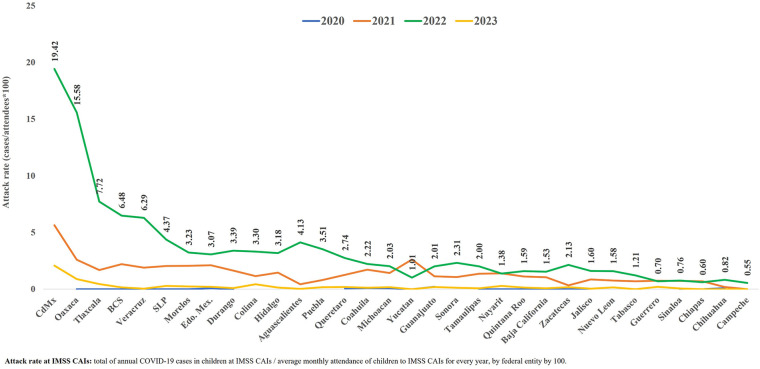
COVID-19 attack rate in children at the IMSS CAIs, by age and federal entity, from July 2020 to March 2023. Source: Epidemiological Surveillance Platform in IMSS Childcare Facilities. March 31st. 2023. Childcare Facilities Information and Administration System (SIAG). //CdMx: Mexico City; BCS: South Baja California; Edo. Mex: State of Mexico; SLP: San Luis Potosi. For the year 2023, information is included up to March 31st.

Analyzing the attack rates by COVID-19 in children from the CAIs by federal entity, it was observed that the highest rates occurred in 2022, except in the states of Yucatan, Nayarit, Guerrero and Chiapas, where the highest rates were reported in 2021. Mexico City had the highest attack rates compared to the rest of the federal entities in 2021–2023 (5.64%, 19.42% and 20.7%, respectively). In 2020, the highest attack rate was reported in the state of Guanajuato (0.20%) (Figure 3).

**Figure 4:**
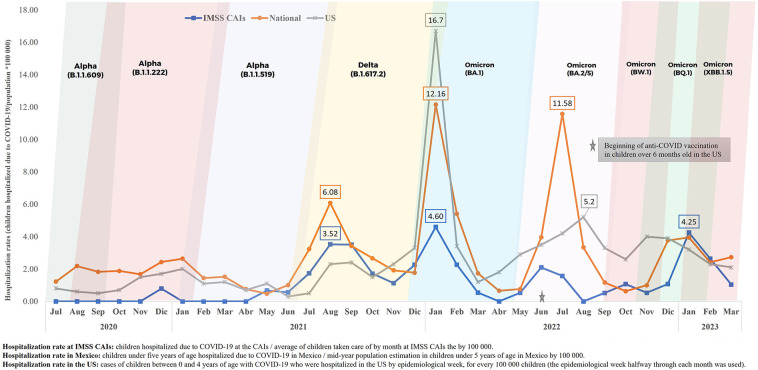
Hospitalization rates due to COVID-19 in children from the IMSS CAIs, at a national level in Mexico and in the US, from July 2020 to March 2023. Source: Epidemiological surveillance platform in IMSS childcare facilities, March 31st, 2023. Childcare Facilities Information and Administration System (SIAG). COVID-19 Open Data, General Direction of Epidemiology (DGE), April 17th, 2023 (20). Mid-year population estimation in children under five years of age in Mexico (2020–2023), General Direction of Health Information (Oct 2022) (21). COVID data tracker, US CDC, June 10th, 2023 (22). COVID-19 variants in 2020–2023 (23, 24).

During the study period, there were four hospitalization incidence peaks due to COVID-19 in children under five years of age at a national level in Mexico, in the US and at the IMSS CAIs: between July and September 2021, between December 2021 and February 2022, between June and August 2022, and between December 2022 and January 2023. As of June 2022 (when anti-COVID-19 vaccination was initiated in children over 6 months of age in the US), Mexico had higher hospitalization rates at a national level compared to the US. During the whole study period, hospitalization rates at a national level in Mexico were higher than at the IMSS CAIs (Figure 4).

**Figure 5:**
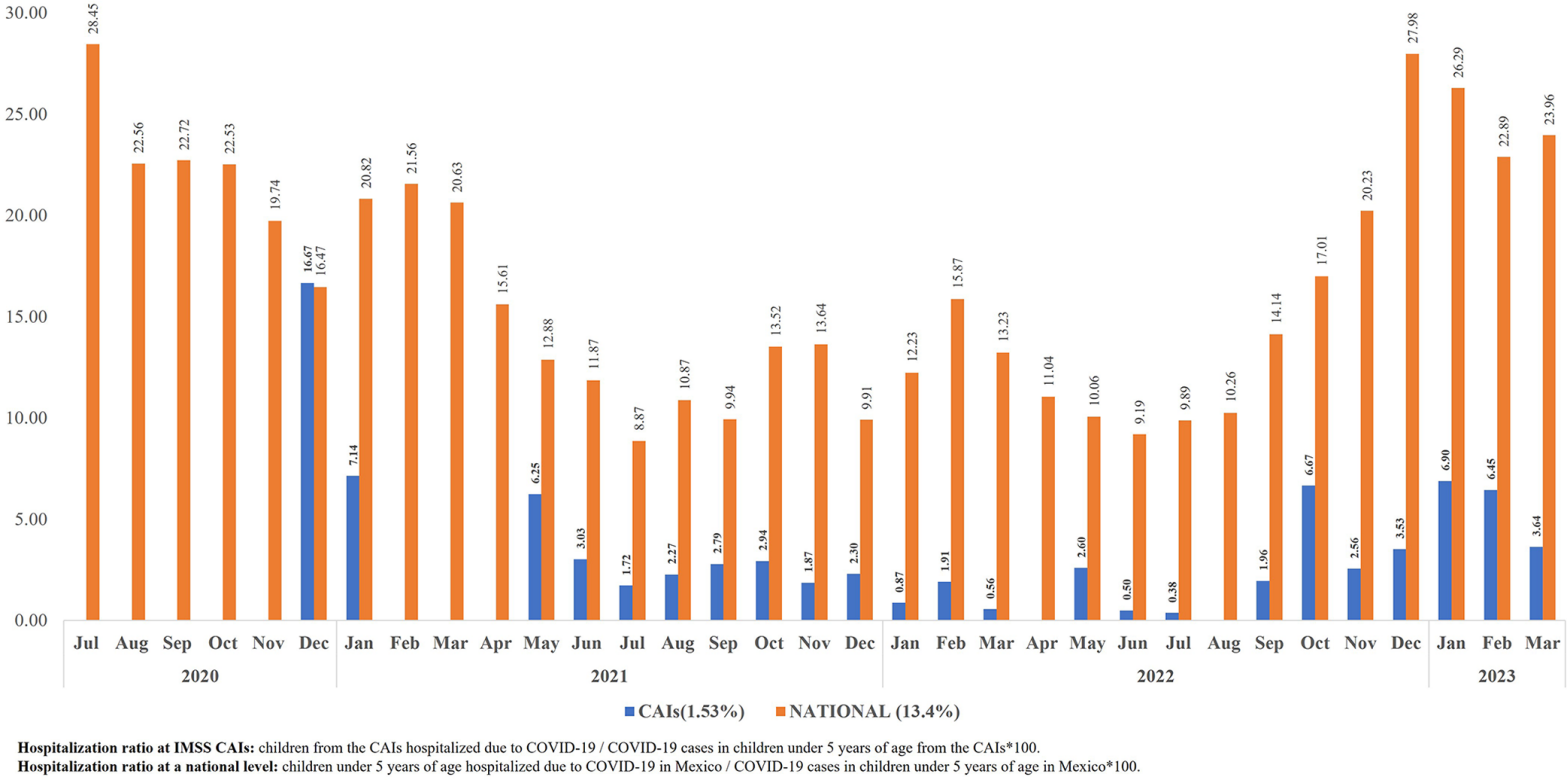
Ratio of children with COVID-19 at the IMSS CAIs and at a national level in Mexico who were hospitalized, from July 2020 to March 2023. Source: Epidemiological surveillance platform in IMSS childcare facilities, March 31st, 2023. COVID-19 Open Data, General Direction of Epidemiology (DGE), April 17th, 2023 (20).

The ratio of children from IMSS CAIs that required hospitalization due to COVID-19 during the study period was 1.53%, in comparison with 13.41% at a national level in Mexico. The higher ratio of hospitalized children with COVID-19 at a national level occurred between July 2020 and March 2021 (prevalence of Alpha variants) and between November 2022 and March 2023 (prevalence of Omicron variants). The highest ratio of hospitalized children with COVID-19 in relation to the total of reported cases at a national level was 28.45% (July 2020), and 16.6% (December 2020) among the children attending the IMSS CAIs (Figure 5).

**Figure 6:**
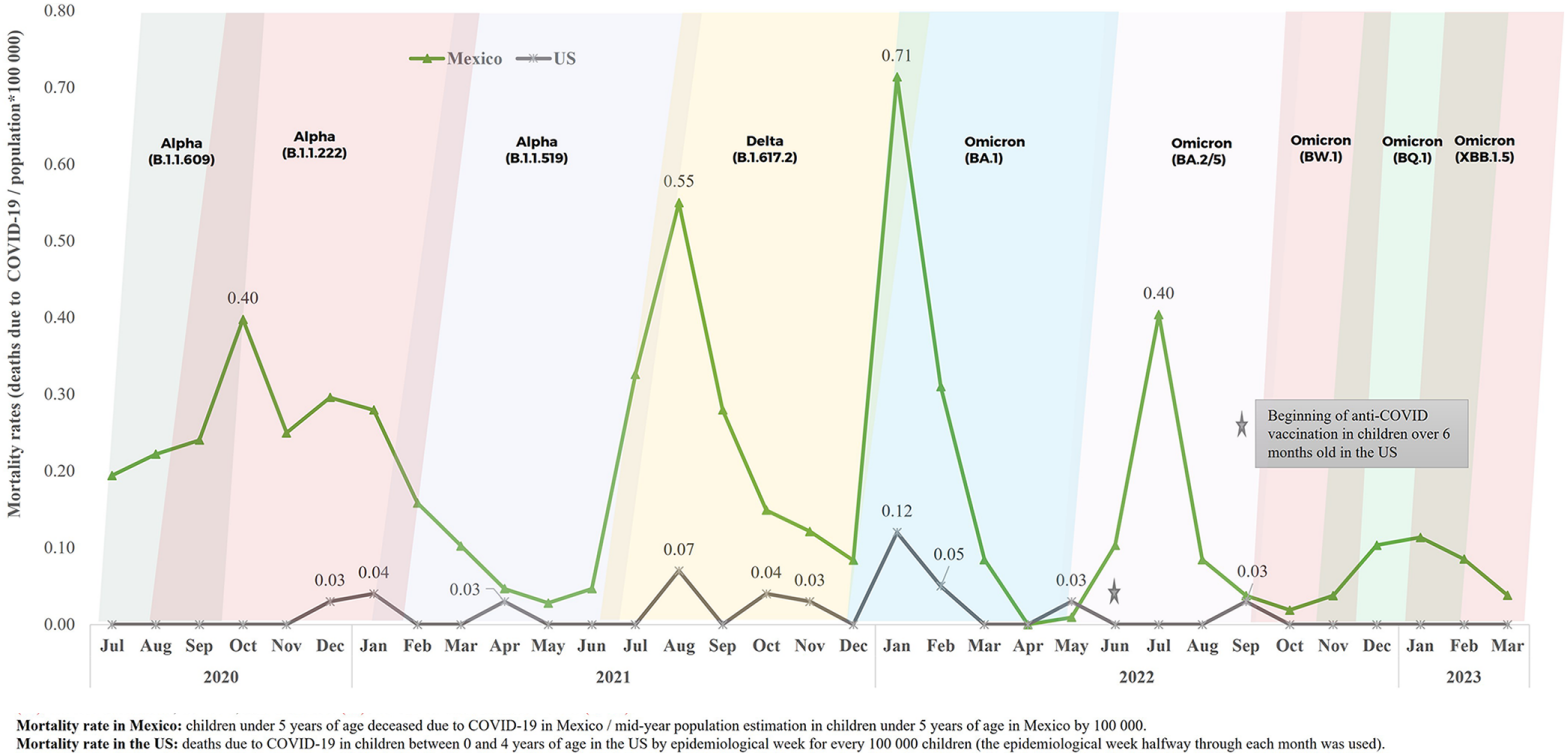
Mortality rate by COVID-19 in children under 5 years of age at a national level in Mexico and in the US, from July 2020 to March 2023. Source: COVID–19 Open Data, General Direction of Epidemiology (DGE), April 17th, 2023 (20). Mid-year population estimation in children under five years of age in Mexico (2020–2023), General Direction of Health Information (Oct 2022) (21). COVID data tracker, US CDC, June 10th, 2023 (22). COVID–19 variants in 2020–2023 (23, 24).

The mortality rate by COVID-19 in children under five years of age in Mexico was higher during the whole study period in comparison to what was reported in the US. In January 2022, the mortality rate in Mexico was 5.9 higher than in the US, which is associated to the emergence of the Omicron BA.1 variant (Figure 6).

Taking as reference the number of cases, hospitalizations and deaths due to COVID-19 in children under five years of age at a national level, it was determined that children taken care of at IMSS CAIs had a lower risk of being hospitalized or dying due to COVID-19 than the rest of the population in this age group. By the year 2020, the children that attended the IMSS CAIs were 77.3% less likely to be hospitalized (RR, 0.22; 95% CI: 0.03–1.53), 80.9% in 2021 (RR, 0.19; 95% CI: 0.12–0.28), 93.2% in 2022 (RR, 0.06; 95% CI: 0.04–0.09), and 77.7% up to March 2023 (RR, 0.23; 95% CI: 0.13–0.38). In 2021, the children that attended the IMSS CAIs were 90.6% less likely to die due to COVID-19 (RR, 0.09; 95% CI: 0.01–0.67), and up to March 2023, this likelihood was 34.3% lower than the rest of the population in this age group (RR, 0.65; 95% CI: 0.08–4.83) (Table 4).

**Table 4:** Risk of disease, hospitalization and death by COVID-19 in children from the IMSS CAIs, from July 2020 to March 2023.

Year	Disease		Hospitalization		Death	
	RR	95% CI	RR	95% CI	RR	95% CI
2020	0.3183	0.2074–0.4886	0.2269	0.0335–1.5377	—^b^	—
2021	2.4164^c^	2.2715–2.5706	0.1908	0.1293–0.2814	0.0942	0.0132–0.6712
2022	3.6191^c^	3.4894–3.7536	0.0679	0.0466–0.0989	—^b^	—
2023^a^	2.7505	2.4096–3.1397	0.2330	0.1398–0.3884	0.6568	0.0892–4.8341

Epidemiological surveillance platform in IMSS childcare facilities, 31st March 2023; COVID-19 Open Data, General Direction of Epidemiology (Dirección General de Epidemiología – DGE), 17 April 2023 (20).

RR, relative risk.

^a^ Confirmed cases are considered between 1st January and 31st March 2023.

^b^ No deaths were reported among the children cared for at the IMSS CAIs during the year reported.

^c^ RR affected by criteria pointed out in the Standardized Guidelines for the Epidemiological Surveillance.

## Discussion

According to the reports of the American Academy of Pediatrics, it was during the emergence of the Delta variant in 2021 (27 June–18 December 2021) when the cases of COVID-19 among children under 17 years of age increased for the first time (25,26). After that, the Omicron BA.1 variant emerged, which caused the greater increase of COVID-19 cases among children under five years of age during the study period. The incidence rate due to this variant in the US was 9 times higher than the one reported in Mexico, which could be explained by several factors: an active epidemiological surveillance of this disease in Mexican medical facilities was carried out mainly in the case of hospitalizations; many ambulatory cases did not look for medical care and were, hence, not reported; the failure to perform diagnostic tests in the ambulatory cases, and the under-reporting of ambulatory cases that did look for medical care. The higher incidence of cases at the IMSS CAIs, compared to the national incidence, is due to the active epidemiological surveillance implemented in all our centers. Likewise, the higher incidence of COVID-19 reported by the CDC in the US is due to a more active epidemiological surveillance (Figure 1).

Regarding the transmission mechanism in schools, there are published studies that suggest that educators play an important role in this process (10). A prospective study of the transmission dynamics of SARS-CoV-2 at schools in the United Kingdom informed that, out of 210 cases related with outbreaks, 154 (73%) occurred among workers and 56 (27%) among children (27). A study carried out in child day-care centers in France reported not having found any evidence of SARS-CoV-2 transmission in the centers (7), and another study in 36 elementary schools in Italy did not report secondary cases among pre-school children (28). In our study, it was not possible to determine the transmission pattern; however, the analyzed information suggests that transmission of the infection among educators is much higher than among children, with an attack rate seven times higher. The lowest attack rates observed among the children at the IMSS CAIs during the whole study period compared to the attack rates among the workers can also be related to the sanitary measures implemented, which account for the lesser exposure of the children population, in particular those under five years of age, who did not receive an anti-SARS-COV-2 vaccine (Figure 2).

The COVID-19 attack rates in children at the IMSS CAIs, estimated from the children that did attend these centers during the pandemic, were substantially higher in 2022. This was the year when all the IMSS CAIs were in operation, with the highest number of children attending, as well as the year when the Omicron variant emerged.

The attack rates were variable among the federal entities in the country, in particular during 2022. This fact could be related with the greater demand for service at the CAIs in some federal entities, a higher population density, a greater exposure to sick people and hence, a higher risk of getting sick, as well as a lesser adherence to the established sanitary security measures. Very low attack rates could be mainly related to a lack of case reporting in the information system (Figure 3).

It is very relevant to highlight the behavior of hospitalizations in Mexico compared to those in the US. Up to October 2021, Mexico had higher hospitalization rates than the ones in the US. Later and up to May 2022, with the emergence of the Omicron BA.1 variant, hospitalization rates in the US increased substantially. After the implementation of vaccination among children over six months of age in that country, hospitalization rates have decreased considerably and consistently (Figure 4).

In Mexico, the highest ratio of children under five years of age with COVID-19 hospitalized in 2020 and at the beginning of 2021, and at the end of 2022 and beginning of 2023, can be mainly due to the type of SARS-COV-2 variant. The circulating virus caused a more serious disease in the children from this age group (Figure 5).

The highest mortality rate due to COVID-19 at a national level in children under five years of age, in comparison to the ones reported by the CDC in the US during the study period, can be related to different factors. Among them, we have the following: late diagnosis of the infection by SARS-COV-2, late search for medical care in serious cases, difficult access to or lack of medical services, lack of social security, and failure to include children in this age group in the anti-SARS-COV-2 vaccination program (Figure 6).

In this study, it was documented that ambulatory cases of COVID-19 and those requiring hospitalization prevailed among males, both at the CAIs and at a national level (Tables 1, 3), similar to previous reports characterizing the disease in children (6, 25, 29). The age groups with more cases of COVID-19 at the CAIs and at a national level were those between 13 and 24 months of age (28% and 26%, respectively) and those between 25 and 36 months of age (30% and 20%, respectively). However, at a national level, 20% of the cases occurred in the group between 49 and 58 months of age. In diverse publications, it is shown that COVID-19 affects all age groups in children, and there is no significant difference in the infection by SARS-CoV-2 in the different age groups (14, 16, 29).

When analyzing the age group of the children hospitalized due to COVID-19, both at the CAIs and at a national level, the great majority (70% and 65%, respectively) were children under 24 months of age. This was similar to what was reported by the CDC in the US, where, up to January 2022, 74.5% of the cases of hospitalization had occurred among children under 24 months of age (26).

At the CAIs, the ratio of hospitalized children under 24 months of age was higher than 30% in 2021 and 2022. During the first three months of 2023, 79% of the cases occurred among children under 13 months of age. Over time, at a national level, the ratio of deceased and hospitalized children under 13 months of age decreased. In contrast, the ratio increased in the group between 13 and 24 months of age. For the whole study period at a national level, it was observed that the higher ratio of deaths (76%) occurred in children under two years of age (Tables 1, 3).

Based on the risk analysis, this study shows that the children that attended the IMSS CAIs during the years 2021 and 2022 and the first quarter of 2023 had a higher risk of getting sick with COVID-19 compared with the rest of the population under five years of age. Even though attending a CAI can expose a child and the workers to a higher risk of getting infected by SARS-COV-2 because the facilities are closed and because it is very hard to prevent direct contact between the children, it is noteworthy that these places carry out an active epidemiological surveillance, which enhances the timely detection of cases. A limiting factor of this analysis is the important under-registration of COVID-19 cases in the official platform of the Ministry of Health for this age group. In contrast, since this platform prioritizes the report of cases of hospitalization and death due to COVID-19 in this age group, the use of these data allowed for an adequate estimation of the risk of hospitalization and death in the children that attend the CAIs (Table 4). The lower risk of hospitalization and death due to COVID-19 (protective factor) observed in the children that attended the IMSS CAIs, compared to the general population under five years of age, is largely due to the fact that these children have social security, which warrants timely referral and medical care.

Another important limiting factor in this study is that the health system in Mexico does not carry out routine tests to detect an infection by SARS-CoV-2 in all children with respiratory symptoms, but prioritizes those with serious disease. This generates an underestimation of the incidence of non-serious cases of COVID-19 at a national level.

## Conclusions

This study suggests that at the IMSS CAIs, as is the case in other child day-care centers and educational institutions in other countries, transmission of SARS-CoV-2 is much higher among workers than among children. The high incidence rates in the children at the CAIs compared to the rest of the children in Mexico point to a national under-registration, contrary to what happens at our CAIs, where timely registration and follow-up of cases is mandatory.

Strict adherence to sanitary safety protocols to prevent and contain the infection by SARS-COV-2 and the fact that most children attended in the IMSS CAIs are generally healthy, and the timely referral from the CAIs to the medical area at IMSS were key factors in the decrease of the risk of hospitalization and death due to COVID-19 in the children cared for in these facilities. However, the high rates of hospitalization and death due to SARS-COV-2 in our country in children under five years of age make vaccination against this virus in this age group necessary and urgent (11, 26, 30).

## Data Availability

Publicly available datasets were analyzed in this study. This data can be found here: https://www.gob.mx/salud/documentos/datos-abiertos-152127.
